# Liver fluke (*Fasciola hepatica*) infection in cattle in Northern Ireland: a large-scale epidemiological investigation utilising surveillance data

**DOI:** 10.1186/s13071-016-1489-2

**Published:** 2016-04-14

**Authors:** Andrew W. Byrne, Stewart McBride, Angela Lahuerta-Marin, Maria Guelbenzu, Jim McNair, Robin A. Skuce, Stanley W.J. McDowell

**Affiliations:** Veterinary Sciences Division, Agri-food and Biosciences Institute (AFBI), Stoney Road, Stormont, Belfast, BT43SD UK

**Keywords:** *Fasciola hepatica*, Meat inspection, Spatial analysis, Northern Ireland, Disease surveillance, Risk factors

## Abstract

**Background:**

Liver fluke (*Fasciola hepatica*) is a widespread parasite of ruminants which can have significant economic impact on cattle production. Fluke infection status at the animal-level is captured during meat inspection of all animals processed for human consumption within Northern Ireland. These national datasets have not been analysed to assess their utility in uncovering patterns in fluke infection at animal- and herd-levels in Northern Ireland.

**Methods:**

We utilised a dataset of 1.2 million animal records from ~18,000 herds across 3 years (2011–2013) to assess animal- and herd-level apparent prevalence and risk-factors associated with fluke infection. Animal-level apparent prevalence was measured as the proportion of animals exhibiting evidence of fluke infection at slaughter; between herd-level infection prevalence was measured by binary categorisation of herds (infected or not). “Within herd” infection prevalence was measured using the proportion of animals within a herd that showed evidence of fluke infection per year (ranging from 0–100 %). “Within herd” infection prevalence at the herd level was investigated using multivariable modelling.

**Results:**

At the animal level, the proportion of animals slaughtered that exhibited evidence of infection was 21–25 % amongst years. Across herds, the proportion of herds with at least one infected animal, varied between 61 and 65 %. However, there was a significant sampling effect at the herd-level; all herds where at least 105 animals slaughtered over the study period exhibited evidence of fluke infection (100 %). There was significant variation in terms of within-herd infection prevalence. Risk factors included herd type, long-term weather variation, geographic location (region) and the abattoir.

**Conclusions:**

Liver fluke apparent prevalence was high at the herd-level across years. However, there was lower prevalence at the animal level, which may indicate significant variation in the exposure to fluke infection within herds. The proportion infected within-herds varied significantly in time and space, and by abattoir, herd-type and some weather variables. These data are a useful source of information on a widespread endemic disease, despite known limitations in terms of test performance (low sensitivity). As well as informing on the distribution and severity of liver fluke infection, these analyses will be used to investigate the effect of co-infection on risk for bovine tuberculosis.

**Electronic supplementary material:**

The online version of this article (doi:10.1186/s13071-016-1489-2) contains supplementary material, which is available to authorized users.

## Background

Bovine fascioliasis, caused by the infection of the liver fluke *Fasciola hepatica*, is a worldwide problem for cattle farming, with infections having impact in terms of mortality losses, reduced weight gain, milk produced and carcass quality [1]. Within Northern Ireland, bovine fascioliasis is a widespread endemic disease of cattle. This is due to Northern Ireland having favourable habitat types and climate for *Galba truncatula*, the main intermediate mollusc host of *F. hepatica.* Furthermore, field based cattle farming is practiced in Northern Ireland with animals being exposed to metacercariae by grazing on pasture*.* While fascioliasis is considered a chronic condition of bovines, bovine mortality attributed to fascioliasis in herds in Northern Ireland has been reported (2–9 % of deaths investigated by state laboratories 2010–2013; [2]). Surveillance data from faecal samples submitted to the Agri-food and Biosciences Institute, Northern Ireland during 2012–2014 showed that between 10 and 17 % of faecal samples contained eggs of *F. hepatica*. Furthermore, with the advent of climate change [3, 4] and the emergence of anthelmintic resistance [5, 6], it is likely that liver fluke may have an even more significant impact on agricultural production in this region [3, 4] and elsewhere [4].

A number of studies have utilised abattoir data to assess infection levels and to model the risk factors and/or spatial distribution of parasitic infections [1, 7–16]. Northern Ireland has a long history of successfully modelling parasitic infections of livestock using surveillance datasets, in particular liver fluke infection [7, 8, 17]; however, recently these data have not been analysed to garner their utility to uncover trends or their use in exploring other epidemiological hypotheses. Recent research from the Republic of Ireland used bulk milk samples from dairy farms to spatially model the distribution, clustering and relative probability of occurrence of liver fluke at a national scale [18–20]. Nevertheless, there has been a lack of similar spatial analyses of the patterns of liver fluke infection risk published across Northern Ireland recently. These data are essential, as fluke burdens carry a significant cost to farmers in terms of production and control, for example, in NI it costs £50 million per annum and ROI €90 million per annum [21]. Furthermore, co-infections of fluke and bovine tuberculosis (caused by *Mycobacterium bovis*) have recently been found to affect the ability to detect bTB exposed animals [22, 23]. Co-infections with other parasites could also be impacting additively to livestock health with the potential for additional economic impacts [16].

The main goal of the present analysis was to assess the relationship between metrics of fluke infection, as measured using abattoir surveillance data, with herd level characteristics and geographically referenced environmental and meteorological predictors. The analysis demonstrates the importance of good surveillance and databasing systems and how these can be utilised for research and policy development. Despite the potential for low animal level sensitivity of abattoir surveillance, aggregating at the herd level allows for useful predictions from a readily available data source [15]. This adds value and understanding to these datasets [13]. Predictions from this work can directly inform further analyses on the potential effects of concurrent infections on the management of other diseases in cattle herds (e.g. bovine tuberculosis in Northern Ireland [24]).

## Methods

### Data

Data were gathered from the Animal and Public Health Information System (APHIS) for Northern Ireland (NI) for all cattle that were slaughtered within abattoirs throughout NI from 2011 to 2013. These data included animal-level (animal identifier, sex, age, bTB status and fluke status), herd-level (herd identifier, herd type, turnover), and geographic variables (region and farm coordinates). More details of the variables used are presented in Additional file 1: Tables S1–S6. All animals were included, with the exception of animals that did not go through an abattoir (e.g. fallen animals that were sent to knackeries; animal carcasses that were deemed not fit for human consumption). All animals processed through abattoirs in Northern Ireland would have been inspected for the presence of fluke infection as part of passive surveillance/meat inspection in compliance with UK and European legislation (EC 854/2004). This legislation requires that all livers from bovines are inspected, and livers may be inspected using visual, incision and palpation inspection for bovines > 6 weeks old [13].

The apparent prevalence was assessed initially using descriptive statistics at animal, within-herd and across-herd levels. The relationship between the number of animals sampled and the estimated prevalence at the herd level was also investigated.

Due to the high levels of fluke exposure across herds (see results), the apparent within-herd prevalence of infection was modelled in relation to risk factors. Prevalence was measured on a scale of 0 to 1, by calculating the proportion of animals within a given year that exhibited evidence of fluke infection. As within-herd prevalence could be affected by the minimum number of samples from each herd, a minimum sample per herd per year of > 12 animals was set [25]. This was based on the estimated mean within-herd sample needed for a sampling strategy using a fixed test cut-point of 1 infected animal and a within-herd prevalence of 20 % [25]. There is a trade-off between increasing the minimum number of animals sampled per herd to increase the robustness of within-herd prevalence estimates, and the number of herds included in a dataset. In the present data set, the mean number of samples per herd was 23.6 (SD 129.2) but the median was 4, indicating a highly screwed distribution. The threshold of > 12 animals, overall across all years, resulted in 45 % of herds being included. On a yearly basis, this proportion varied between 37 and 40 %. Separate models were developed for each year of the study, 2011, 2012, and 2013, respectively.

Summary statistics of the independent variables are described in the Additional file 1: Tables S1–S6. These variables were selected based on availability and literature searches (e.g. [3, 15, 23, 26]). Herd size (based on the number of animals tested during a full TB herd test), region (the District Veterinary Office (DVO) area; 10 administrative areas), herd type (five broad categories) were all derived from the APHIS dataset. “Herd type” was based on the recorded enterprise type within APHIS, five categories were formed, including beef breeding, beef fattening, beef rearing, dairy, and “other”. The “other” category included dealers (*n* = 29 herds), exporters (*n* = 11) and unclassified or mixed herds (*n* = 4,672). A variable called “turnover” was also included, which was calculated as the number of animals slaughtered in a given year, divided by the recorded herd-size. The majority of herds slaughtered a proportion of their herd size in a given year (i.e. turnover < 1). However, some herds had a greater number of animals slaughtered than the recorded herd size (i.e. turnover > 1). This arose due to high turnover in certain herds, and the fact that herd size was estimated from one static annual recording (the annual bovine tuberculosis test) at a given point during the year. Therefore, turnover captured both the buying in and slaughtering of cattle (high turnover herds have to repopulate their herds to maintain stock).

A geographic information system (GIS; ArcGIS) was used to extract data for the herds within our dataset. The coordinates for each farm (point location of herd as registered within APHIS) within the dataset were used to spatially associate the location of each cattle herd during its last ordinary herd before being sent to slaughter. With these location data, we were able to assign land use (CORINE or the UK land classification) and climatic (long-term monthly weather trends) to each farm at a 5 km grid scale. Official data was gathered from the UK Meteorological Office (www.metoffice.gov.uk) using the publicly available 5 km grid datasets (United Kingdom Climate Impact Programme (UKCIP) 5 km^2^ datasets). Long-term monthly data on the mean values over 1981–2010 for maximum and minimum monthly temperatures, the mean number of rain days (>1 mm) per month and humidity was used; an approach used to model *F. hepatica* previously in the UK [3] and Ireland [19]. We also gathered Vapour Pressure data, however the long-term mean monthly values available for the region only extended from 1971–2000. The mean temperature (derived from the minimum and maximum data) and climatic variance (standard deviation around the mean of each long-term monthly variable) was included. For each month, the long-term monthly mean was extracted, and then averaged these values and calculated how much variation there was in terms of the standard deviation (SD) around the mean. Land cover variables (based on CORINE and the UK land cover map 2000 [27]) were also used as a cofactor, as landscape composition has been shown to affect fluke prevalence in cattle (e.g. [26]). The CORINE categories were compressed into eight broad land types: arable, complex cultivation patterns, discontinuous urban fabric, good pasture, mixed pasture, poor pasture, mixed Agriculture/natural, and other landscape types. For a land type to be a separate category, at least 1 % of herds had to be associated with the land class type. All other rarer land types were aggregated into the category called “other landscape types”. An alternative land cover dataset was also explored; UK land cover categories into competing models, with the following categories included in the variable: acid grass, arable horticulture, calcareous grass, dense dwarf shrub heath, improved grassland, inland bare ground, neutral grass, open dwarf shrub heath, suburban/rural developed, and “other”.

### Modelling

Within herd prevalence was modelled in two ways. Firstly, severity was dichotomised into a binary outcome (0/1), where “0” herds exhibited within-herd yearly prevalence in the lower 25 %ile of the overall distribution; “1” herds exhibited within herd yearly prevalence in the upper 25 %ile of the overall distribution (note, this approach does remove farms with levels of infection between the 25^th^ and 75^th^ percentile). This outcome was modelled using a multivariable logit model, with independent variables presented in Additional file 1: Tables S1–S6. The fit of the model to the data was assessed using a Hosmer-Lemeshow goodness-of-fit test (calibration); *P* ≤ 0.05 indicated significant lack of fit to the data. The discriminatory ability of the model was assessed using the Area Under the Curve (AUC) from a Receiver Operated Curve (ROC). The AUC is measured on a continuous scale from 0 to 1; an AUC of 0.5 is no better than random, > 0.7 is considered an adequate model, > 0.8 is a good model, and > 0.9 is considered an excellent model. The sensitivity (probability that a positively classified farm is truly positive), specificity (probability that a negatively classified farm is truly negative), and the percentage of farms that were correctly classified, using a standard cut-point of 0.5 was reported too. Assessment of whether there was any spatial autocorrelation in the residuals of the final models was assessed by plotting a semi-variogram using the package geoR [28] within the R statistical environment [29]. A 95 % confidence envelope was created by randomly permuting the data across the locations 99 times (i.e. assuming no spatial autocorrelation) following [23] and then plotting the envelope within which 95 % of the semivariances lay [28]. The observed and predicted outcomes from the models were mapped using ArcGIS® (version 9.2; Redlands, CA: Environmental Systems Research Institute), with spatial variation presented using Inverse Weighted Distance (IWD) interpolation (Fig. 2).

The second modelling approach was to model the within-herd prevalence as a continuous outcome (proportion/fraction) using Fractional Response Regressions [30] and Generalised Linear Modelling. The benefit of this modelling approach is that all the data can be used (as opposed to the binary approach used above), and the expected outcome (fraction positive per year) can be modelled as a continuous variable while bounding the outcome between O and 1 (i.e. between 0 and 100 % positive), in comparison with an ordinary least squares (OLS) model [31]. The models were developed using Generalised Linear Model (GLM) modelling with the proportion infected as the outcome using a logit link function and robust standard errors and compared the results with those of the fractional response regression. We compared the predictions from these models against the observed proportion infected, but binning the predicted proportions in categories (groups of 0.05, respectively) and visually assessing the relationship (calibration graph; [32, 33]).

During model building, all independent variables were tested for association with the outcome variable using univariable models (either logistic regressions or GLMs). The functional form of the relationship between the outcome variable and continuous predictors was assessed using LOWESS smoothed curves. Where necessary, independent variables were suitably transformed [34]. Predictors were retained for the multivariable model if associated with the outcome at *P* < 0.2. Throughout, model building was undertaken using backwards, forwards and stepwise sequential model building [34]. Correlations between independent variables were assessed, and Variance Inflation Factor (VIF) was used during model building to assess if multi-collinearity may have been a problem (using OLS models). The final models were compared to assess their composition, and Akaike’s information criteria (AIC) were used if there were competing final models. The best models were those with the lowest AIC value. All statistical analyses were undertaken in Stata 14 (StataCorp 2015, College Station, TX).

## Results

### Descriptive analysis

Overall, there were 1,216,579 animal level records within the dataset (Table 1). Of these, 288,114 (23.68 %; 95 %CI: 23.61–23.76 %) animals were recorded with signs of fluke infection. The percentage of animals with fluke varied across years, with higher apparent prevalence in 2011 (24.80 %) and 2013 (24.85 %), than 2012 (21.37 %). This difference between 2012 and other years was statistically significant (2012 *vs* 2011: OR: 0.82; *P* < 0.001; 2012 *vs* 2013: OR: 0.82; *P* < 0.001; *n* = 1,216,579), while there was no difference between 2011 and 2013 (*P* = 0.619; *n* = 1,216,579).

**Table 1:** Animal level variation in *post-mortem* fluke infection status across years (2011–2013) in Northern Ireland

Animal fluke status	2011	2012	2013	Total
No	311,891	316,109	300,465	928,465
(%)	75.20	78.63	75.15	76.32
Yes	102,862	85,905	99,347	288,114
(%)	24.80	21.37	24.85	23.68
Total	414,753	402,014	399,812	1,216,579

The mean within-herd percentage positive overall was 23.18 % (median 14.86 %; IQR: 0–35.53; Table 2). The within-herd percentage varied across years; varying from 24.60 to 21.38 % from 2011–2013 (Table 2).

**Table 2:** The mean percentage of animals within-herds that exhibited evidence of liver fluke infection

	2011	2012	2013	Mean
Mean within-herd %	24.60 %	21.38 %	23.55 %	23.18 %
Std. Dev.	0.281	0.270	0.274	0.276
Total herds	13,907	13,655	12,435	13,332

The herd-level fluke infection status was assessed for each herd-year and for each herd respectively. In total, there were 39,997 herd-years within the dataset, of which 25,259 herd-years (63.15 %) were positive for fluke (where at least one animal with fluke found within the herd-year). This varied over time, between 64.84 and 60.85 % during 2011–2013 (Table 3). Aggregating across years, there were 18,266 herds represented in the dataset, with 12,621 herds having evidence of fluke infection within their herd over the study period (69.10 %; 95%CI: 68.42–69.77 %). However, there was a significant sampling effect found, with a relationship between the number of animals sampled, and the probability of being an exposed herd (see next section).

**Table 3:** Herd level variation in fluke infection status, with herds categorised as exposed (with at least one infected animal culled in a given year) or non-exposed (without any culled animals with evidence of fluke infection at *post-mortem*) across years (2011–2013) in Northern Ireland

Herd fluke status	2011	2012	2013	Total
Non-Exposed	4,890	5,346	4,502	14,738
(%)	35.16	39.15	36.20	36.85
Exposed	9,017	8,309	7,933	25,259
(%)	64.84	60.85	63.80	63.15
Total	13,907	13,655	12,435	39,997

### Relationship between numbers of animals sampled and herd status

**Fig. 1:**
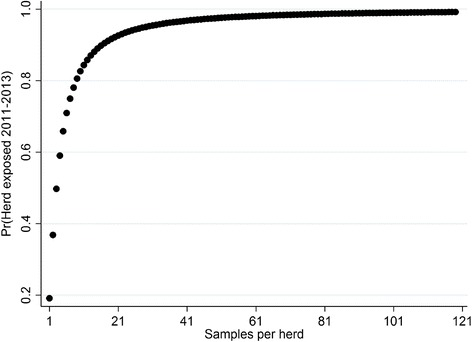
The relationship between the probability of a herd having evidence of fluke infection and the number of animals slaughtered per herd in Northern Ireland 2011–2013

There was a significant relationship between the numbers of animals sampled (culled within an abattoir) per herd across years 2011–2013, and the probability of that herd having a positive exposed status (log(no. samples) OR: 3.68; *P* < 0.001; Table 4). Herd-level percentage positive increased from 34.22 % for herds which had between one and four animals culled between 2011 and 2013, up to 100 % positive for herds with 105 or more animals submitted to abattoir over the study period (Table 4). The relationship between apparent prevalence and sampling was steep (Fig. 1), with 95 % of herds with 20–39 animals sampled being positive for fluke infection. This indicates that effectively all herds (with a large numbers of animals slaughtered to aid detectability) in Northern Ireland were exposed to fluke (this could be either home bred or bought in animals) over a temporal resolution of three years.

**Table 4:** The overall status of herds in NI relative to the number of samples taken per herd over the period 2011–2013. Note, the cut-point where there were no fluke free herds was 105 animals sampled

Herd status				Samples				
	1–4	5–9	10–19	20–39	40–79	80–159	>160	Total
0	4,535	709	272	93	31	5	0	5,645
%	65.78	30.36	12.97	4.70	1.62	0.32	0	30.91
1	2,359	1,626	1,825	1,887	1,878	1,560	1,485	12,620
%	34.22	69.64	87.03	95.3	98.38	99.68	100	69.09
Total	6,894	2,335	2,097	1,980	1,909	1,565	1,485	18,265

Similar patterns were found when status was assessed on individual years. There was a significant relationship between the number of samples per herd per year and the % herds considered exposed in 2011 (log (no. samples) OR: 3.54; *P* < 0.001), 2012 (OR: 3.32; *P* < 0.001), and 2013 (OR: 3.35; *P* < 0.001). All herds which culled > 160 animals in 2011, > 227 in 2012 and > 302 in 2013 respectively, were fluke positive (Table 5).

**Table 5:** The yearly status of herds in NI relative to the number of samples taken per herd for the years 2011–2013, respectively. Note, the cut-point where there were no fluke free herds was 160 animals sampled in 2011, 227 in 2012, and 302 in 2013

Herd status (2011)				Samples				
	1–4	5–9	10–19	20–39	40–79	80–159	>160	Total
0	4,019	543	225	93	7	3	0	4,890
%	64.46	28.16	12.14	5.26	0.59	0.53	0	35.16
1	2,216	1,385	1,629	1,676	1,188	562	361	9,017
%	35.54	71.84	87.86	94.74	99.41	99.47	100	64.84
Total	6,235	1,928	1,854	1,769	1,195	565	361	13,907
Herd status (2012)								
	1–4	5–9	10–19	20–39	40–79	80–159	>160	Total
0	4,255	600	325	127	35	3	1	5,346
%	69.91	31.88	17.74	7.35	2.94	0.52	0.28	39.15
1	1,831	1,282	1,507	1,602	1,155	576	356	8,309
%	30.09	68.12	82.26	92.65	97.06	99.48	99.72	60.85
Total	6,086	1,882	1,832	1,729	1,190	579	357	13,655
Herd status (2013)								
	1–4	5–9	10–19	20–39	40–79	80–159	>160	Total
0	3,508	560	289	98	36	7	4	4,502
%	68.68	32.60	16.71	5.58	3.04	1.24	1.07	36.2
1	1,600	1,158	1,441	1,657	1,148	559	370	7,933
%	31.32	67.40	83.29	94.42	96.96	98.76	98.93	63.8
Total	5,108	1,718	1,730	1,755	1,184	566	374	12,435
Grand total negative	11,782	1,703	839	318	78	13	5	14,738
Grand total positive	5,647	3,825	4,577	4,935	3,491	1,697	1,087	25,259
Grand total	17,429	5,528	5,416	5,253	3,569	1,710	1,092	39,997

### Multivariable logit model results

The binary logit outcome model categorised high and low prevalence (referred to as “severity”) herds, respectively based on the being within the top quartile of all herds or being in the bottom quartile of all herds (0/1). The cut-point for high-risk herds was average within-herd prevalence of > 33.33 % (coded as “1”); the cut-point for the lower risk herds was < 11.03 % (coded as “0”). This resulted in 7,746 total herds, 3,997 (51.60 %) high risk herds and 3,749 (48.40 %) low risk herds across the 3 years of this study.

### 2011 model

The final 2011 logit model contained herd severity estimates for 2,472 herds, and exhibited a pseudo-R^2^ (McFadden’s) of 0.28. The discriminatory ability of the model was good (AUC: 0.82). Apparent sensitivity was 84.78 %, specificity was 67.82 %, and 77.47 % of herds were correctly classified according to the predictions of the model. The model fitted the data well (Hosmer-Lemeshow test: *P* > 0.05). There was no evidence of spatial autocorrelation within the residuals from the final model.

The final model suggested that there was significant variation in risk across abattoirs, herd types and DVO regions (Table 6). There was a large difference in the risk amongst abattoirs; for example, the odds ratio for abattoir 1 relative to abattoir 0 was 10.79 (*P* < 0.001). This equated to 35 % of herds associated with abattoir 0 being in the high risk group, whereas 83 % of herds associated with abattoir 1 were in the high risk group. Dairy herds had the highest risk of being in the high severity category across herd types (OR: 1.65 relative to beef breeding; *P* < 0.001); 66 % of dairy herds were in the high risk category in comparison to a mean of 51 % across other herd types. Beef fattening herds had significantly lower risk of being classified as a high severity herd relative to beef breeding (OR: 0.71; *P* < 0.05). Fluke risk was negatively associated with increasing long-term minimum temperature (*P* < 0.001). Herds within the regions of Derry, Armagh, Larne (County Antrim) and Omagh (County Tyrone) had the lowest odds of being high severity herds; whereas herds in Ballymena (County Antrim) and Enniskillen (County Fermanagh) had the highest probability of being high severity herds.

**Table 6:** Outcomes from three multivariable logit models relating the risk of cattle herd exhibiting high severity fluke infection for the years 2011, 2012 and 2013, respectively

Logit		2011	*N* = 2,472	2012	*N* = 2,408	2013	*N* = 2,500
Predictor		OR	Sig.	OR	Sig.	OR	Sig.
Abattoir	0	1		1		1	
	1	10.790	***	9.231	***	16.595	***
	2	5.021	***	4.735	***	2.720	***
	3	8.209	***	4.551	***	11.665	***
	4	0.221	***	0.093	***	0.195	***
	5	0.483	**	0.419	**	0.397	***
	6	1.438		0.894		1.054	
	7	3.725	***	6.927	***	15.264	***
Rain days	mean_rain			1.536	***		
SD Rain days	sd_rain					0.513	*
Max. temp.	maxtemp			1.573	*		
Min. temp.	mintemp	0.630	***			0.574	***
Humidity	humidity					0.701	*
Herd type	Beef breeding	1		1		1	
	Beef fattening	0.713	*	0.950		0.736	
	Beef rearing	0.943		0.684		0.814	
	Dairy	1.651	***	2.185	***	1.062	
	Other	1.102		1.197		0.544	***
Region	Armagh	1		1		1	
	Ballymena	1.810	*	6.192	***	5.069	***
	Coleraine	1.191		2.367	***	3.333	***
	Derry	0.832		1.158		1.423	
	Dungannon	1.274		2.066	**	1.021	
	Enniskillen	1.602		1.566		1.850	
	Larne	0.910		3.390	***	3.948	***
	Newry	1.043		1.526		1.194	
	Newtownards	1.142		1.629	*	2.136	***
	Omagh	0.699		1.136		1.830	

Significance (Sig.): * *P* < 0.05; ** *P* < 0.01; *** *P* < 0.001

**Fig. 2:**
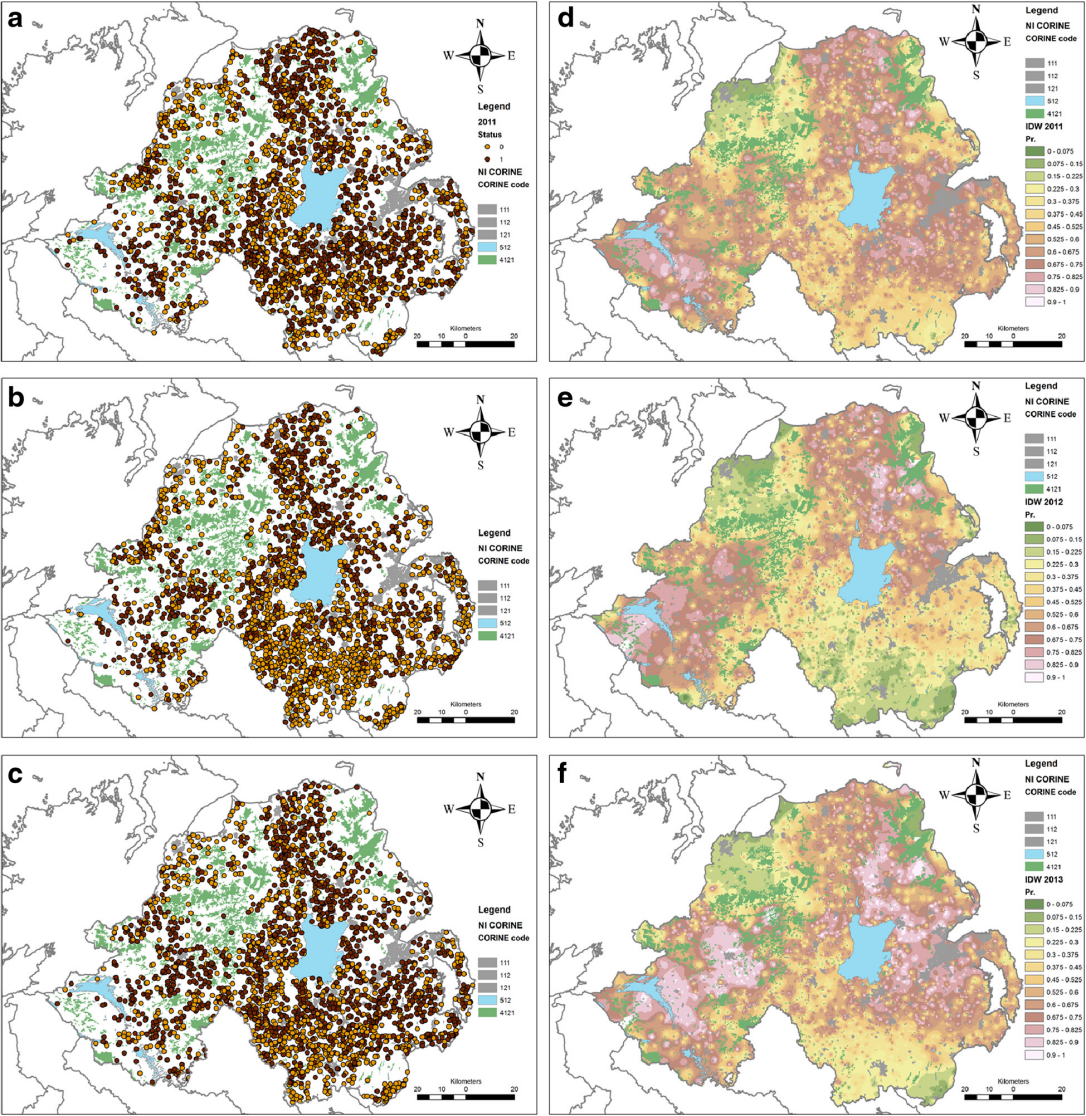
Observed and predicted within-herd liver fluke prevalence in Northern Ireland based on meat inspection data. Panels **a**-**c** depicts the distribution of herds with high or low within-herd infection prevalence for 2011–2013, respectively. Panels **d**-**f** represents predicted probability of a herd being in a high infection category from 2011–2013 from a logistic model, respectively. The CORINE land cover types presented in this map corresponds with: 111 = Continuous urban fabric; 112 = Discontinuous urban fabric; 121 = Industrial and commercial units; 512 = Water bodies; 4121 = Unexploited bogs

The distribution and status of farms making up this dataset is presented in Fig. 2a. Predictions from the logit model are presented in Fig. 2d. The distribution of these herds is widespread across Northern Ireland, with some notable exceptions in North Tyrone/South Derry, North-East Antrim, South-East Down, and the greater Belfast area (see Additional file 1: Figures S3 and S4). These areas correspond to marginal or unproductive lands (upland, and areas with bog) and urban areas (Fig. 2a).

### 2012 model

The final multivariable logit model contained 2,408 herds, with a pseudo-R^2^ of 0.32. The discriminatory ability of the model was very good (AUC: 0.86) and exhibited apparent sensitivity of 76.64 %, specificity of 79.55 %, and correct classification of 78.32 %. The model fitted the data well (Hosmer-Lemeshow test: *P* > 0.05), and there was no evidence of spatial autocorrelation in the residuals from the final model.

A risk map derived from the model’s predictions in presented is Fig. 2e, and compared to observed point maps derived from observed data (Fig. 2b). Fluke severity risk varied significantly amongst the predominant abattoir used, herd-type and region (Table 6). Herds predominately using abattoirs 1 (OR: 9.23) and 7 (OR: 6.93) were of considerably higher risk of being a high risk herd relative to herds using abattoir 0 (*P* < 0.001). In 2012, 24 % of herds using abattoir 0 were in the higher prevalence category, whereas 69 and 77 % of herds using abattoir 1 and 7 were high risk respectively. Dairy herds again were the highest risk amongst herd types (OR: 2.19 relative to beef breeding; *P* < 0.001); 58 % of dairy herds were in the high prevalence category, whereas the average proportion across the other categories was 33 %. Fluke severity risk was also positively associated with increasing long-term mean maximum-temperature (*P* = 0.011) and with increasing number of rain days (*P* < 0.001).

### 2013 model

The final 2013 multivariable logit model contained 2,500 herds and had a pseudo-R^2^ of 0.33. The model exhibited very good discriminatory ability with an AUC of 0.86. Using a probability cut-off of 0.5, the model exhibited sensitivity of 82.97 %, specificity of 77.12 % and was able to correctly classify 80.40 % of herds. Residuals from the final model did not exhibit spatial autocorrelation (Additional file 1: Figures S1 and S2). A risk map derived from the model’s predictions in presented in Fig. 2f.

Variation in the probability of a herd having a high fluke infection severity score varied significantly by the predominant abattoir, herd type, region, long-term variation in rain fall (SD), mean humidity and minimum-temperature (Table 6). Dairy herds had the highest risk of exhibiting high severity fluke infection relative to beef or other herd types. Armagh (south-east region) had the lowest risk, while Ballymena (north-east region) had the highest risk of being in the severe fluke category. The risk of severe fluke infection decreased with the increasing variation in rainfall, minimum temperature and humidity.

### Proportion infected model

Both the GLM and the fractional response regression models produced very similar results. Consequently, the GLM models are presented here only. The outcomes from the models for 2011, 2012 and 2013, respectively, are presented in Table 7. The ability of the model to capture the relative within-herd prevalence was assessed by plotting the predicted prevalence against the observed prevalence. The calibration of the models was generally good, with some evidence of the models marginally overestimating within-herd prevalence at low infection levels, and underestimating the mean within-herd prevalence at the high infection levels (see Additional file 1: Figures S11–S13).

**Table 7:** Generalised linear models (GLM) relating the proportion of animals with evidence of fluke infection at *post-mortem* within-herds for the years 2011, 2012 and 2013 respectively. The GLM was fitted with a logit link function to ensure predictions from the model would be constrained between 0 and 1 (i.e. 0 to 100 % infected within herd)

GLM		2011	*N* = 4,680	2012	*N* = 4,794	2013	*N* = 4,874
Predictor		OR	Sig.	OR	Sig.	OR	Sig.
Abattoir	0	1		1		1	
	1	1.953	***	2.158	***	2.014	***
	2	1.652	***	1.798	***	1.307	***
	3	1.817	***	1.777	***	1.911	***
	4	0.613	***	0.636	***	0.586	***
	5	0.791	**	0.979		0.758	**
	6	1.110		1.086		0.926	
	7	1.442	***	2.005	***	1.936	***
Herd size	herdsize					0.971^	***
Min. temp.	mintemp					0.885	***
SD min. temp.	SD mintemp			0.777	*		
SD humidity	SD humidity	1.074	**	1.088	**		
Humidity	humidity			0.931	*		
Herd type	Beef breeding	1		1		1	
	Beef fattening	0.919	*	0.938		0.917	*
	Beef rearing	0.978		0.921		0.960	
	Dairy	1.098	**	1.188	***	1.059	
	Other	1.011		1.028		0.940	
Region	Armagh	1		1		1	
	Ballymena	1.229	***	1.585	***	1.374	***
	Coleraine	1.139	**	1.379	***	1.359	***
	Derry	1.025		1.273	*	1.148	
	Dungannon	1.093		1.195	***	1.032	
	Enniskillen	1.318	***	1.438	***	1.156	*
	Larne	1.001		1.390	***	1.373	***
	Newry	1.034		1.086		0.982	
	Newtownards	1.074		1.120	*	1.296	***
	Omagh	1.041		1.317	***	1.064	

^ = per 100 animals; Significance (Sig.): * *P* < 0.05; ** *P* < 0.01; *** *P* < 0.001

Across models, abattoir, region and herd type were significant predictors of within-herd apparent prevalence. Abattoir 1 was consistently associated with the highest proportion of animals with evidence of fluke infection across years (OR: 1.95–2.16 relative to abattoir 0; *P* < 0.001). Abattoir 4 was consistently associated with the lowest proportion of animals with evidence of fluke infection across years (OR: 0.59–0.64 relative to abattoir 0; *P* < 0.001). Dairy herds exhibited a higher proportion of animals with evidence of fluke infection relative to other herd types (OR: 1.06–1.19 relative to beef breeding herds; *P* < 0.01: 2011–2012; *P* < 0.1: 2013). Ballymena, Coleraine and Enniskillen had significantly higher within-herd prevalence (*P* < 0.05; Table 7) relative to Armagh which had the lowest within-herd prevalence. Herd size was negatively associated with increasing within-herd prevalence only during 2013 (OR: 0.97 per 100 animals; *P* < 0.001).

Across years, different climatic variables were associated with within-herd prevalence. In 2011 and 2012, within-herd prevalence was positively associated with increasing long-term variation in humidity (OR: 1.07–1.09; *P* < 0.01). In 2012, fluke within-herd prevalence was also associated with decreasing humidity (OR: 0.93; *P* < 0.05) and lower variation in minimum temperatures (OR: 0.78; *P* < 0.05). In 2013, there was an association with the long-term minimum temperature and within-herd fluke prevalence (OR: 0.89; *P* < 0.001).

## Discussion

Our study has demonstrated that there are high levels of liver fluke exposure at animal and herd levels in Northern Ireland. Overall, between 21 and 25 % of all animals were recorded with evidence of fluke infection across the years of the study. A similar level of fluke infection from an abattoir from northern Portugal/Spain was reported, where 28 % of 776 animals slaughtered were infected with liver fluke [35]. However, the levels of fluke infection reported at abattoir are lower than previous *post-mortem* studies undertaken in the Republic of Ireland [12] and Northern Ireland [36]. Murphy et al. [12] reported that 65 % of livers from culled cows that were laboratory inspected had fluke or pathology attributable to fluke infection. In Northern Ireland, an abattoir survey in 1964–1965 found that 88 % of cattle had been infected with *F. hepatica* at some stage of their life [36]. This reflects the fact that routine surveillance can have low sensitivity to detect infection. A study by Rapsch et al. [37] suggested that meat inspection for liver fluke may exhibit a sensitivity of 63.2 % (55.6–70.6 %), meaning that the true levels of infection may be between 1.5 and 2 times the apparent prevalence. In Switzerland, abattoir surveys suggested that the prevalence of fluke infection in cattle was between 8.4 and 15% [38–40]. However, accounting for test sensitivity, the true prevalence in a study of 1,331 animals was 18.0% (95 % credible intervals 15.9–20.3 %), which indicated that approximately a third of infected livers were missed by meat inspection during that study [37]. It should be noted, however, estimates of the sensitivity of routine meat inspection in Northern Ireland is currently unknown.

Importantly, the levels of infection reported here are considerably higher than reports from meat inspection data from Denmark presented in Olsen et al. [15], where only 3–4 % of animals were deemed infected per annum. Similarly, in Sweden meat inspection data suggested that animal level prevalence was 3 % in 2005, but rose to 9.8 % in 2012 and 11 % in 2013 (reported in [41, 42]). These international differences are possibly the result of a true difference in the exposure to liver fluke between the countries [4, 42]. The island of Ireland is possibly the best suited region within Europe for liver fluke to thrive, both in terms of climate and habitat suitability, as highlighted by recent international modelling efforts [4, 43, 44]. Furthermore, climate projections suggest the weather on the island of Ireland will be more favourable for fluke in the future [3, 4]. Warmer, wetter winters will ensure survival of fluke metacercariae and their intermediate host within the Irish landscape, increasing risk of transmission. Furthermore, the advent of flukicidal resistance is an emerging issue having a negative impact on the control of fluke infection [5, 6].

At herd level, 61–65 % of all herds had some evidence of fluke infection in the current study. However, we have shown that there was a strong sampling effect with this estimate (Fig. 1). Between-herd prevalence for herds where only 1–4 animals were culled was between 30 and 36 %. The apparent prevalence increased quickly with sample numbers, with apparent prevalence increasing to 82–88 % when 10–19 animals were sampled per herd per annum. For large herds with many animals’ culled (> 105), the herd-level prevalence reached 100% across the years of the study. These results concur broadly with recent prevalence studies in Ireland that suggest that 82 % (*n* = 3,764) of the dairy herds sampled exhibited evidence of exposure to fluke infection (assessed by testing bulk milk samples; [18]). These studies indicate that the vast majority of herds in Ireland have been exposed to liver fluke infection (which could be as a result of exposure on farm, or buying in infected animals) in contrast to other European countries [4, 41, 43]. For example, using similar data (meat inspection) to the present study herd-level between farm prevalence in Denmark has been reported as between 25 and 29 % [15]. In the Netherlands, in a study of 2,500 farms, 30.2 % exhibited evidence of liver fluke exposure (seropositive animals present; [4, 45]). Reports from Belgium found 37 % of herds seropositive [46], in Portugal 11–42 % [47] and in Germany 24 % positive [48]. The seroprevalence of fluke in cattle herds in Sweden has been reported as being 21–29 % [41, 42]. Interestingly, during that Swedish study 17 % of herds that were sero-positive did not have evidence of infection in animals at slaughter, highlighting the poor sensitivity of meat inspection [41, 42].

### Temporal and spatial trend

Over the study period, there was no clear increasing or decreasing trend in apparent prevalence of liver fluke infection. Instead, metrics of infection at animal, within-herd, and between herd levels, suggested that 2012 had significantly lower infection levels than either 2011 or 2013. A contemporary study of fluke infection levels in sheep in Ireland suggested also that there was not significant inter-annual variation in fluke prevalence over a similar period [44].

There was significant variation in risk depending on the region (DVO area) in which the farm resided. Across models, there was greater likelihood of herds being higher risk if they were found in Ballymena or Coleraine, relative to other DVOs. After model building, we did not find evidence of spatial autocorrelation amongst model residuals, in comparison with randomised variograms. This suggests that most of the residual spatial information was captured by the fixed effects in the model. There were a number of fixed effects that were spatially explicit, that may have helped to control for spatial variation, including the REGION fixed effects.

### Dairy herd’s higher risk

Across models, we consistently found that there was higher risk of fluke infection in dairy herds than in non-dairy herd types. Dupuy et al. [13] used cluster analysis to group variables from meat inspection data, to assess its utility in syndromic surveillance in France. The authors found that liver fluke infection was associated with beef cattle. However, liver fluke infection also clustered with female cows and older cows, both of which are characteristic of dairy cattle herds. Murphy et al. [12] reported high levels of polyparasitism in Irish beef and dairy cattle; however, they did not present evidence of a clear herd-type bias. In Denmark, Olsen et al. [15] found that 35 % of non-dairy herds had evidence of fluke infection, compared to 58 % of dairy herds. However, their final model reported that medium sized dairy (30–100 animals) herds had a lower risk of being infected (53 %) with liver fluke than non-dairy herds (56 %) using meat inspection data. In the present study, no interactions between herd-size and type-was found across models (*P* > 0.1 across models). The actual mechanism as to why dairy herds tended to have higher within-herd prevalence is yet to be determined, but could be related to the frequency, timing and type of anthelmintics used, the age profile of the herds, differences in habitats or the grazing management amongst herd types.

### Inter-abattoir variation

One of the most significant factors affecting the variation in fluke prevalence in the present study was the main abattoir used by the farmer over the study period. The average proportion of animals disclosed as fluke positive could be twice as high in one abattoir relative to another abattoir in a given year. Similar variation between slaughterhouses has been reported from Denmark [15]. There, herds associated with the highest disclosing abattoir were 3.4 times more likely to be infected (62 %) than the lowest disclosing abattoir (18 %; [15]). In the present study, across all models, abattoir 1 was associated with herds with high prevalence, while abattoir 4 had the lowest proportion positive. However, the relative rank of other abattoirs varied somewhat across years and model types. The variation in abattoir is related to the performance of meat inspection and the region in which the abattoir was based. While generally animals would be processed in abattoirs close to the herd, there was variation in this pattern across Northern Ireland. When looking at the relationship between abattoir and region, there were some weak correlations found, however Variance Inflation Factor (used in OLS models) was low. A correlation matrix between region and abattoir across the study period suggested that there were some farmers from each DVO sending animals to each of the respective abattoirs. This is maybe linked to the variation in prices achieved across the market.

Previous research from Ireland has found significant variation in the disclosure by meat inspection of bovine tuberculosis (lesions) from slaughter houses [49, 50]. Frankena et al. [49] found that there was a large difference in the rates of bTB like lesions across 42 different factories ranging from 0 to 58 per 10,000 animals slaughtered during 2003–2004. A follow-up survey from 2005–2007 suggested that the variation across factories reduced between surveys (a seven-fold difference to a five-fold difference in disclosure) and the mean proportion disclosed increased [50], indicating that there was scope for improvements in standardisation and the ability to increase detection probabilities [51]. Collins [52] suggested that variation in the performance between abattoirs may be related to a number of factors including line speed, light intensity, inspector’s experience and motivation [52]. Other issues may relate to variation in the actual recording (data quality) of fluke infection between abattoirs. The large variation between abattoirs cannot easily be explained currently, and may be an important future research topic in Northern Ireland.

### Long-term weather correlated with fluke infection but not robust patterns

We found that long-term climate variables were correlated with within-herd prevalence; however, there was large variation in the weather variables selected after modelling building across years and models (Tables 6 and 7). Across three of the models, there was a significant negative association between increasing minimum temperature and liver fluke prevalence. This would indicate that areas that were cooler than average may have increased risk of having higher within-herd prevalence, which is in keeping with larger scale studies [43]. However, in one model, there was a positive association between mean maximum temperature and within-herd fluke prevalence. Such variation in association between weather variables and metrics of fluke prevalence has been reported previously in a study in England and Wales [26]. Surprisingly, variation in rainfall was retained as a predictor in only two models in the current study. In those models, there was an increased risk of higher prevalence with increasing rainfall days, and a decreased risk with the long-term variation in rainfall days, respectively. A recent study comparing fluke infection in sheep between Ireland, Switzerland and Italy found that longer-term weather variables (temperature and rainfall) explained between country variations in fluke prevalence, but were not associated with year-to-year variation within country [44]. Instead, shorter-term weather variation in mean temperature, rainfall and seasonality was important in explaining the variation from year to year within country [44]. McCann et al. [26] found that both mean 5-year and contemporaneous weather variables were associated with fluke exposure in herds in England and Wales. However, there was only a marginal benefit to using a 5-year mean with contemporaneous weather, than using a single time point alone.

At the univariable level, we found some evidence of variation in fluke prevalence depending on the landscape/land cover categories derived from either CORINE or the Land Classification Map. However, neither broad landscape/land cover categories were retained in any of our final models. This concurs with work undertaken Ducheyne et al. [43], who modelled fluke spatial distribution across Europe using weather and vegetation indices. The authors reported that weather variables were very important predictors in their models; however the vegetation variables were not important. McCann et al. [26] found weather variables were consistently important predictors of fluke prevalence in England and Wales, but also found that landscape features were also important for some models. For example, they found the improved grassland was a positive predictor of fluke infection in four of their models. In Switzerland, soil conditions and forest cover was also used to create indicative map of fluke risk, based on the potential exposure of herds to the intermediate host *Galba truncatula* [37]. Other authors have highlighted how the lack of detail of land cover maps may reduce the accuracy of generating risk maps for liver fluke [53]; indeed high resolution mapping may help to elucidate the risk to individual farms and their landscape composition.

### Limitations

The farm locations were only measured using point locations of the farmstead. This has obvious accuracy issues (see [54] for a discussion on positional error in epidemiological studies), as farms may expand out across a landscape and is often associated with a number of farm parcels. However, research has shown that in large scale epidemiological investigations, the farm location can perform well as a practical indicator of farm location [55]. Durr & Fraggatt [55] investigated a number of point geo-referencing systems (centroids, post-codes, farm building location) for farms in Cornwall, England to find the single “best” point location. Overall, centroids performed best in terms of identifying with the largest area of the farm; however the authors judged the farmstead location was the overall best practical point location. The authors highlight the benefits of using the farmstead as it can often be close to the focal point for housed animals over winter, and a centre of activities such as calf rearing and milking. Despite this, Durr & Fraggatt [55] highlight how using the farmstead underperforms where there is extensive numbers of land parcels associated with a farm, which is often the case in Northern Ireland.

The long-term weather datasets may reflect climate variation across Northern Ireland, rather than shorter term weather variation that may drive fluke prevalence. When using *post-mortem* meat inspection data, we are uncertain when during the life of the animal were they infected with fluke. The mean age of animals was 3.2 years (95^th^ percentile: 12.6 years old), with the oldest animals being born in 1997. Therefore, we used the aggregated climate data, but future studies may benefit from reducing the cohort to younger animals and associating the outcome with weather variables over shorter temporal periods. Previous studies have used both long-term and recent weather variables to predict fluke risk, with both longer- and shorter-term datasets being useful depending on the attributes of the data and the period over which they were collected [19, 26].

## Conclusion

The study has shown that fluke infection levels are high in Northern Ireland at animal- and herd- levels, as assessed by *post-mortem* meat inspection data. The majority of herds in Northern Ireland had some animals infected with liver fluke; however, there was significant variation in within-herd prevalence. There was significant spatial variation in risk of herd having high within-herd prevalence, which was associated with variations in long-term weather variables, herd enterprise type and the abattoir processing the animals. We have demonstrated that despite the limitations of using meat inspection data (for example, low test sensitivity and not being able to assign period of exposure to positive animals), they provide a significant cost-effective epidemiological resource that may have been underutilised. The analyses presented will provide useful information for further testing of epidemiological hypotheses in Northern Ireland (e.g. the effects of concurrent infection on disease disclosure).

## Additional file

Additional file 1: Summary of predictor variables **(Table S1-S5)**, example of spatial distribution of residuals and semi-variogram **(Figures S1-S2)**, distribution and prediction maps **(Figures S3-S10)**, and calibration graphs for GLM models **(Figure S11-S13)**. (DOCX 6797 kb)
