# Supplementary material for: Liver fluke (Fasciola hepatica) infection in cattle in Northern Ireland: a large-scale epidemiological investigation utilising surveillance data
Source: Parasit Vectors. 2016 Apr 14;9:209. doi: 10.1186/s13071-016-1489-2 (PMC4832448; doi:10.1186/s13071-016-1489-2)

**Additional file 1**

**Table S1:** Summary of herd related independent predictors (herd size and herd turnover) in herds with animals slaughtered in Northern Ireland during 2009-2011

| **Variable** | **Observations** | **Mean** | **Std. Dev.** | **Min** | **Max** |
| --- | --- | --- | --- | --- | --- |
| **Log(herd size)** | 18,266 | 3.908 | 1.119 | -2.303 | 7.499 |
| **Log(turnover)** | 18,223 | -2.176 | 1.342 | -5.817 | 5.116 |

**Table S2:** Summary of long-term weather related variables for study herds in Northern Ireland 2009-2011.

| **Variable** | **Obs** | **Mean** | **Std. Dev.** | **Min** | **Max** | **Unit** | **Explanation** |
| --- | --- | --- | --- | --- | --- | --- | --- |
| **Humidity** | 16,233 | 83.141 | 0.531 | 79.917 | 84.500 | % | Mean monthly relative humidity |
| **SD(Humidity)** | 16,233 | 2.996 | 0.483 | 1.193 | 4.097 | % | Mean monthly relative humidity |
| **Rain days** | 16,233 | 13.871 | 1.337 | 10.833 | 18.500 | days | Mean rain days >1mm per month |
| **SD(Rain days)** | 16,233 | 1.685 | 0.319 | 0.953 | 2.462 | days | SD(Mean rain days per month) |
| **Max. Temp** | 16,233 | 12.081 | 0.551 | 9.083 | 13.250 | Degree Celsius | Mean max. Temp per month |
| **SD(Max. Temp)** | 16,233 | 4.395 | 0.167 | 3.579 | 4.697 | Degree Celsius | SD(Mean max. Temp per month) |
| **Min. Temp** | 16,233 | 5.115 | 0.470 | 3.545 | 7.273 | Degree Celsius | Mean min. Temp per month |
| **SD(Min. Temp)** | 16,233 | 3.725 | 0.157 | 3.233 | 4.059 | Degree Celsius | SD(Mean min. Temp per month) |
| **Vapour pressure** | 16,233 | 9.335 | 0.161 | 8.333 | 10.167 | hPa | Mean monthly VP |
| **SD(Vapour pressure)** | 16,233 | 2.312 | 0.062 | 1.992 | 2.558 | hPa | Mean monthly VP |

**Table S3:** Tabulation of land classes for study herds in Northern Ireland 2009-2011.

| **Land classification** | **Freq.** | **Percent** |
| --- | --- | --- |
| **Acid grass** | 1,282 | 7.9 |
| **Arable horticulture** | 1,273 | 7.84 |
| **Calcareous grass** | 658 | 4.05 |
| **Dense dwarf shrub heath** | 232 | 1.43 |
| **Improved grassland** | 10,207 | 62.87 |
| **Inland Bare Ground** | 87 | 0.54 |
| **Neutral grass** | 1,158 | 7.13 |
| **Open dwarf shrub heath** | 322 | 1.98 |
| **Suburban/rural developed** | 666 | 4.1 |
| **Other** | 350 | 2.16 |
| **Total** | 16,235 | 100 |

**Table S4:** Tabulation of land classes for study herds in Northern Ireland 2009-2011.

| **CORINE** | **Freq.** | **Percent** |
| --- | --- | --- |
| **Complex cultivation patterns** | 2,384 | 14.69 |
| **Discontinuous Urban Fabric** | 191 | 1.18 |
| **Good pasture** | 6,935 | 42.72 |
| **Mixed Agriculture/natural** | 664 | 4.09 |
| **Mixed pasture** | 4,390 | 27.04 |
| **Non Irrigated arable land** | 261 | 1.61 |
| **Poor pasture** | 767 | 4.72 |
| **Other** | 641 | 3.95 |
| **Total** | 16,233 | 100 |

**Table S5:** Tabulation of herd types for study herds in Northern Ireland 2009-2011.

| **Herd type** | **Freq.** | **Percent** |
| --- | --- | --- |
| **Beef breeding** | 6,630 | 36.30 |
| **Beef fattening** | 1,462 | 8.00 |
| **Beef rearing** | 1,834 | 10.04 |
| **Dairy** | 3,625 | 19.85 |
| **Other** | 4,715 | 25.81 |
| **Total** | 18,266 | 100 |

**Table S6:** Tabulation of district veterinary office (DVO) regions for study herds in Northern Ireland 2009-2011.

| **DVO** | **Freq.** | **Percent** |
| --- | --- | --- |
| **Armagh** | 1,879 | 10.3 |
| **Ballymena** | 1,168 | 6.4 |
| **Coleraine** | 2,160 | 11.84 |
| **Derry** | 734 | 4.02 |
| **Dungannon** | 2,169 | 11.89 |
| **Enniskillen** | 1,945 | 10.66 |
| **Larne** | 1,350 | 7.4 |
| **Newry** | 2,942 | 16.13 |
| **Newtownards** | 1,612 | 8.84 |
| **Omagh** | 2,281 | 12.51 |
| **Total** | 18,240 | 100 |


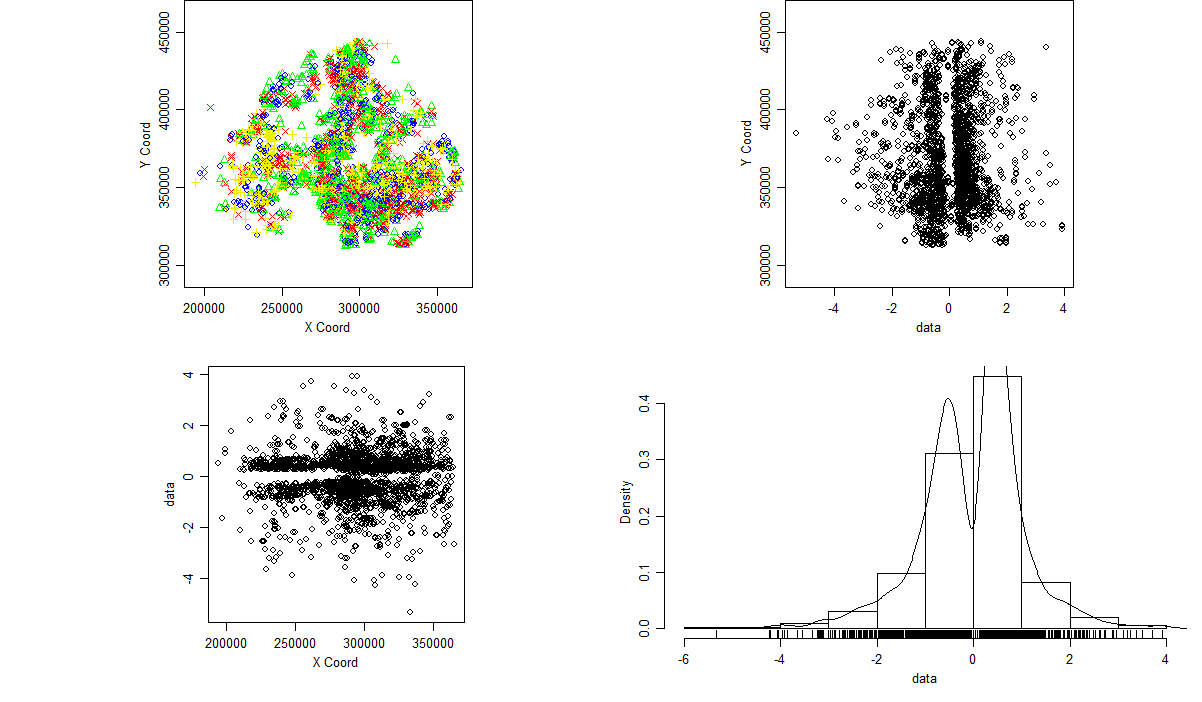


**Figure S1:** Residuals from the final 2013 binary logit model in relation to their frequency and spatial distribution.


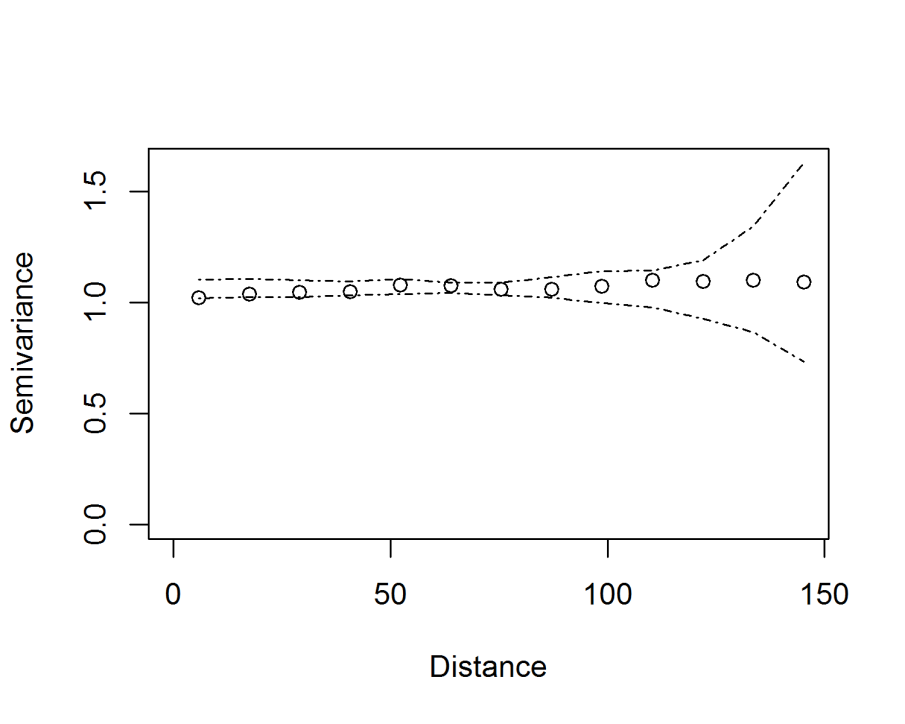


**Figure S2:** Semivariogram of the spatial autocorrelation within residuals from a binary logit model 2013. Distance is measured in km; the dashed lines represent the semivariance produced from empirical variograms computed from 99 independent permutations of the residuals over the locations


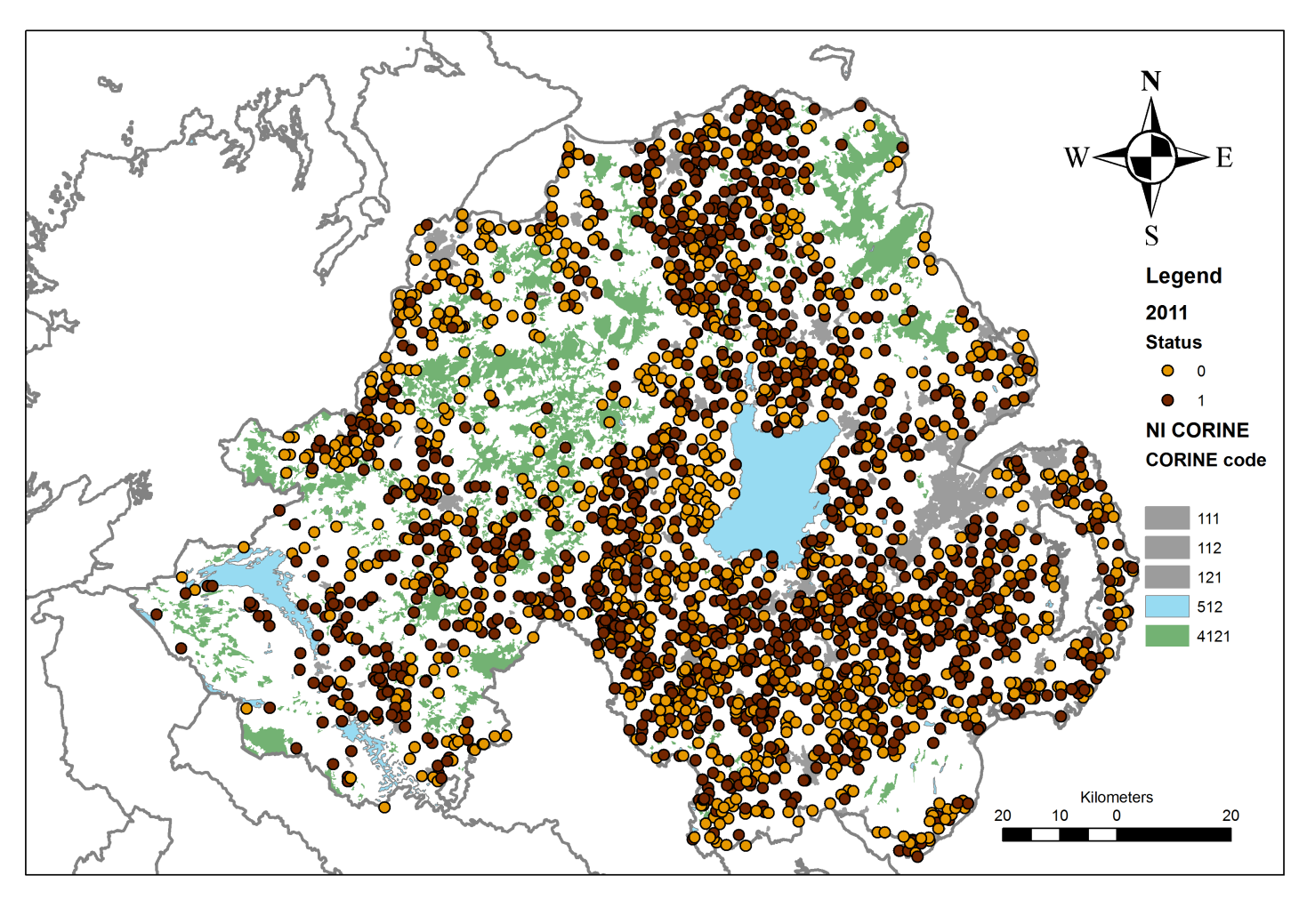


**Figure S3:** Point map of the severely infected (status = 1) farms and the non-severely fluke infected farms (status = 0) in Northern Ireland in 2011. The CORINE land cover types presented in this map corresponds with: 111=Continuous urban fabric; 112 = Discontinuous urban fabric; 121 = Industrial and commercial units; 512 = Water bodies; 4121 = Unexploited bogs.


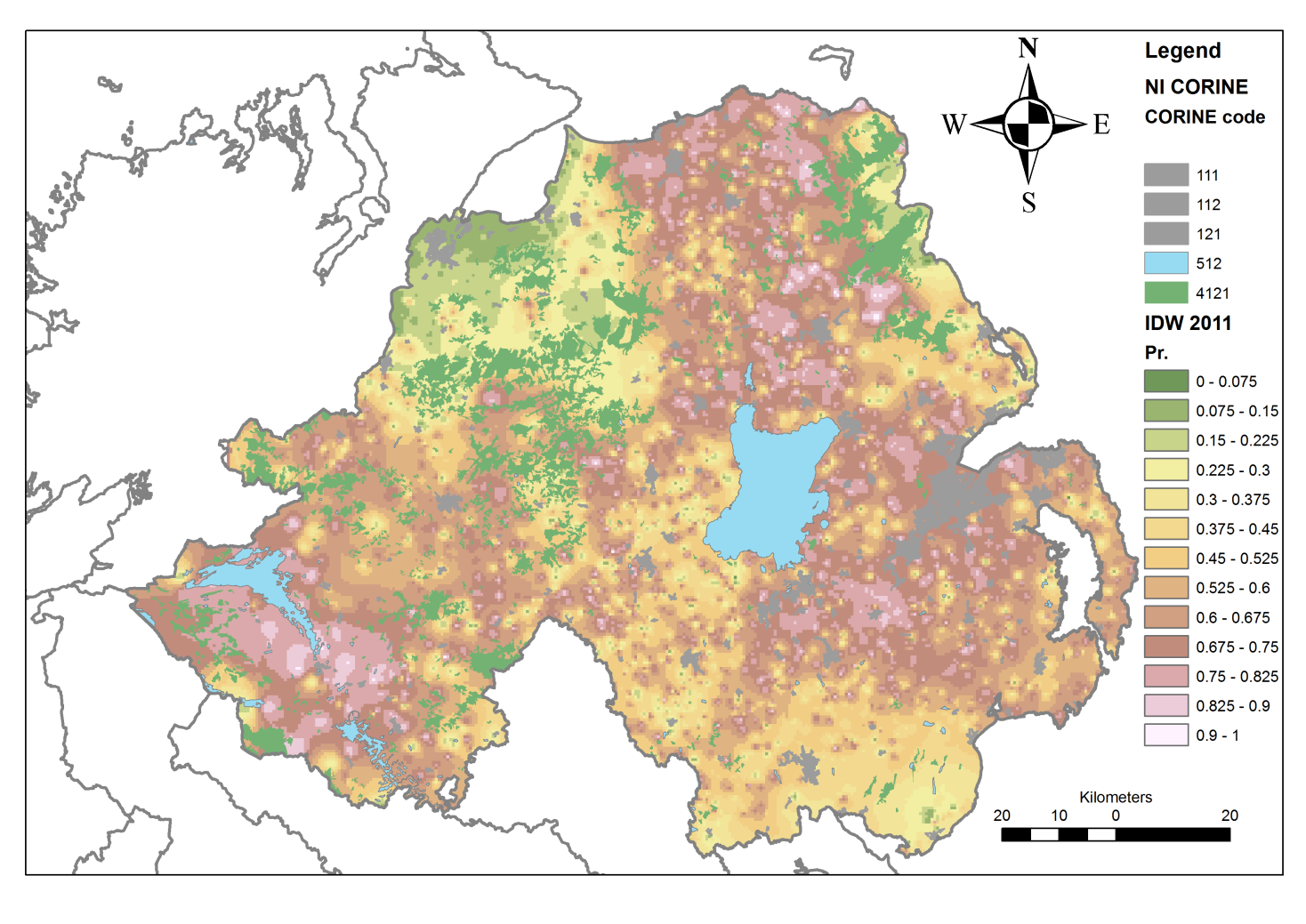


**Figure S4:** Raster map of the predicted within herd prevalence in Northern Ireland in 2011. The CORINE land cover types presented in this map corresponds with: 111=Continuous urban fabric; 112 = Discontinuous urban fabric; 121 = Industrial and commercial units; 512 = Water bodies; 4121 = Unexploited bogs.


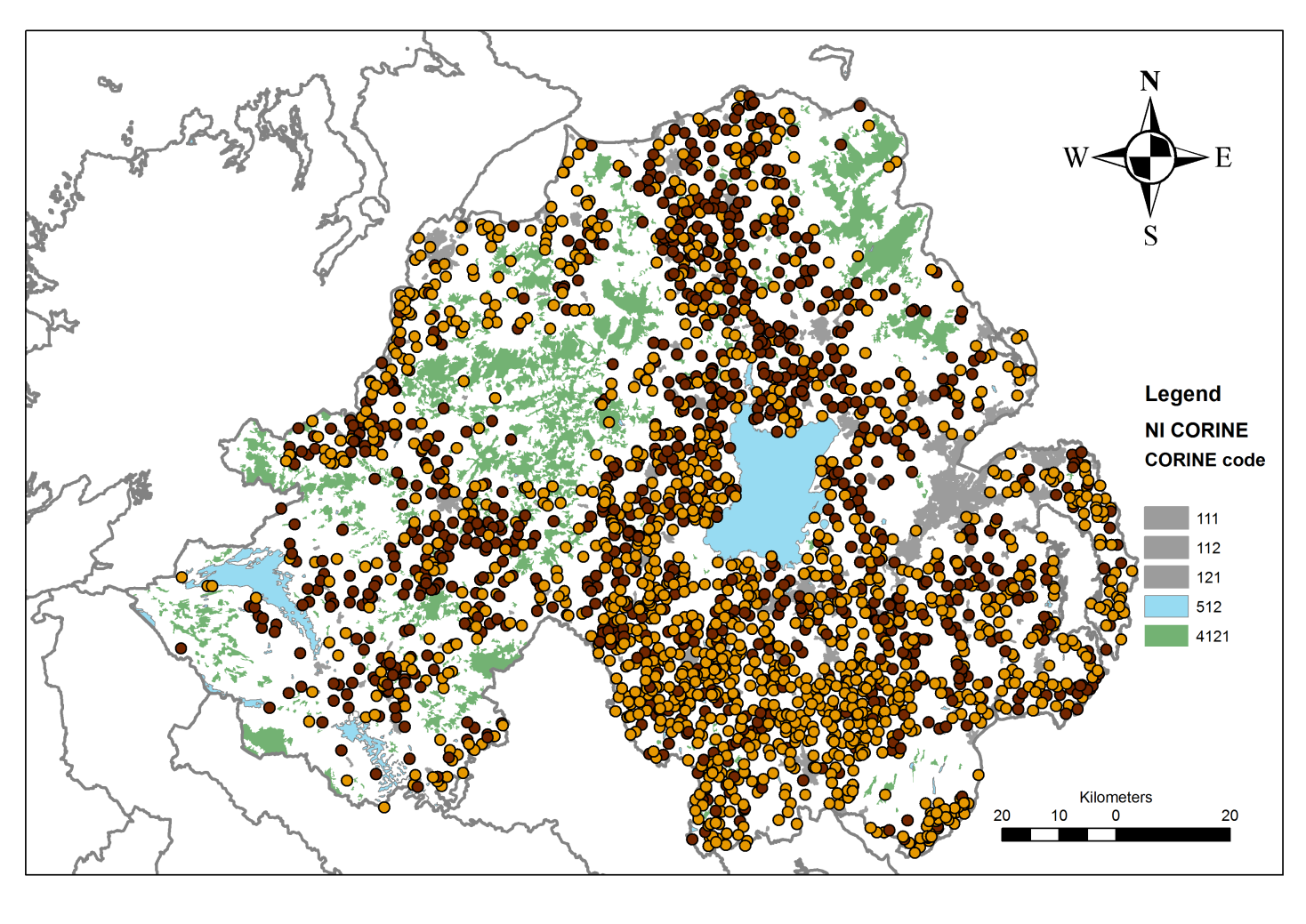


**Figure S5:** Point map of the severely infected (status = 1) farms and the non-severely fluke infected farms (status = 0) in Northern Ireland in 2012. The CORINE land cover types presented in this map corresponds with: 111=Continuous urban fabric; 112 = Discontinuous urban fabric; 121 = Industrial and commercial units; 512 = Water bodies; 4121 = Unexploited bogs.


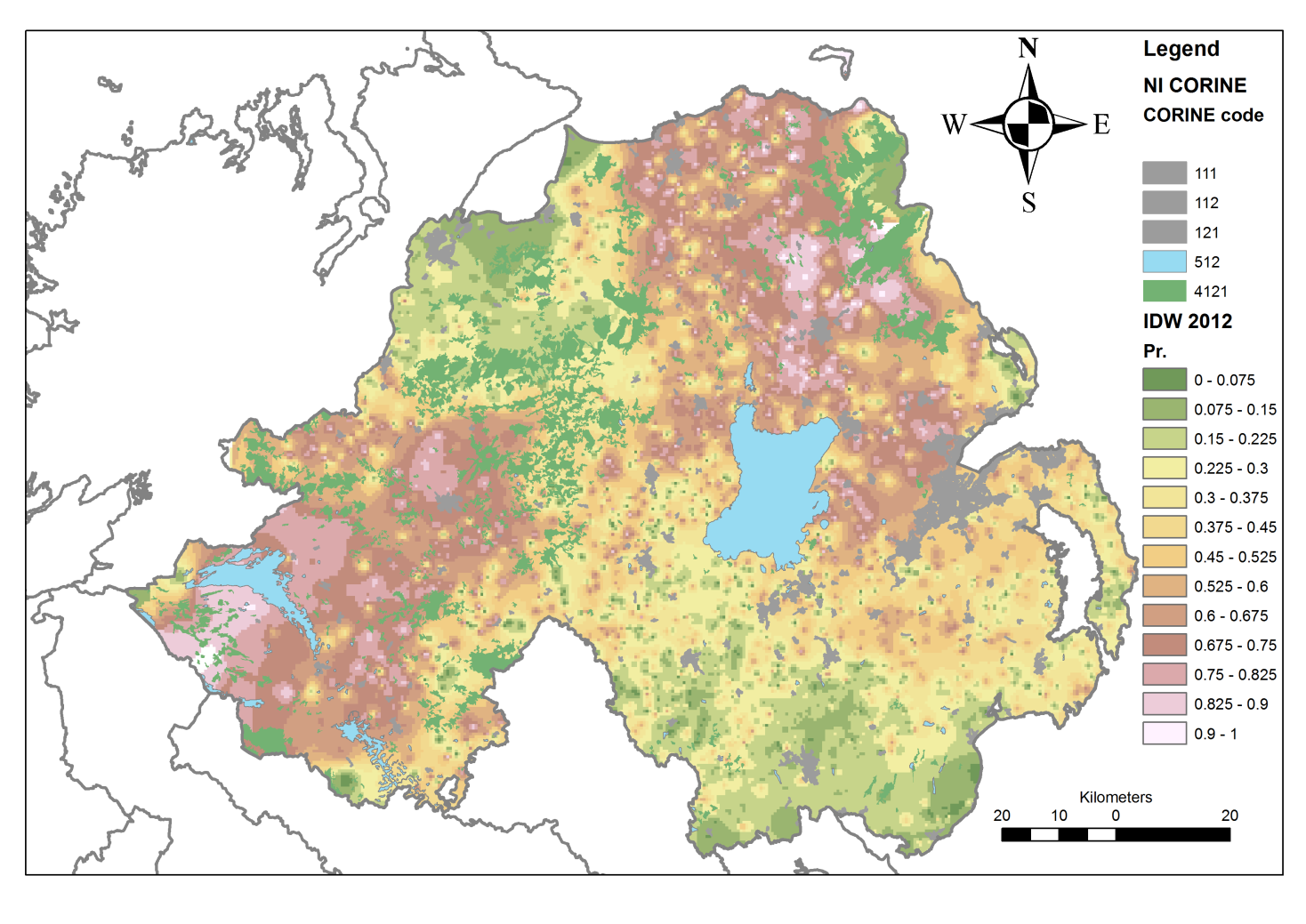


**Figure S6:** Raster map of the predicted within herd prevalence in Northern Ireland in 2012. The CORINE land cover types presented in this map corresponds with: 111=Continuous urban fabric; 112 = Discontinuous urban fabric; 121 = Industrial and commercial units; 512 = Water bodies; 4121 = Unexploited bogs.


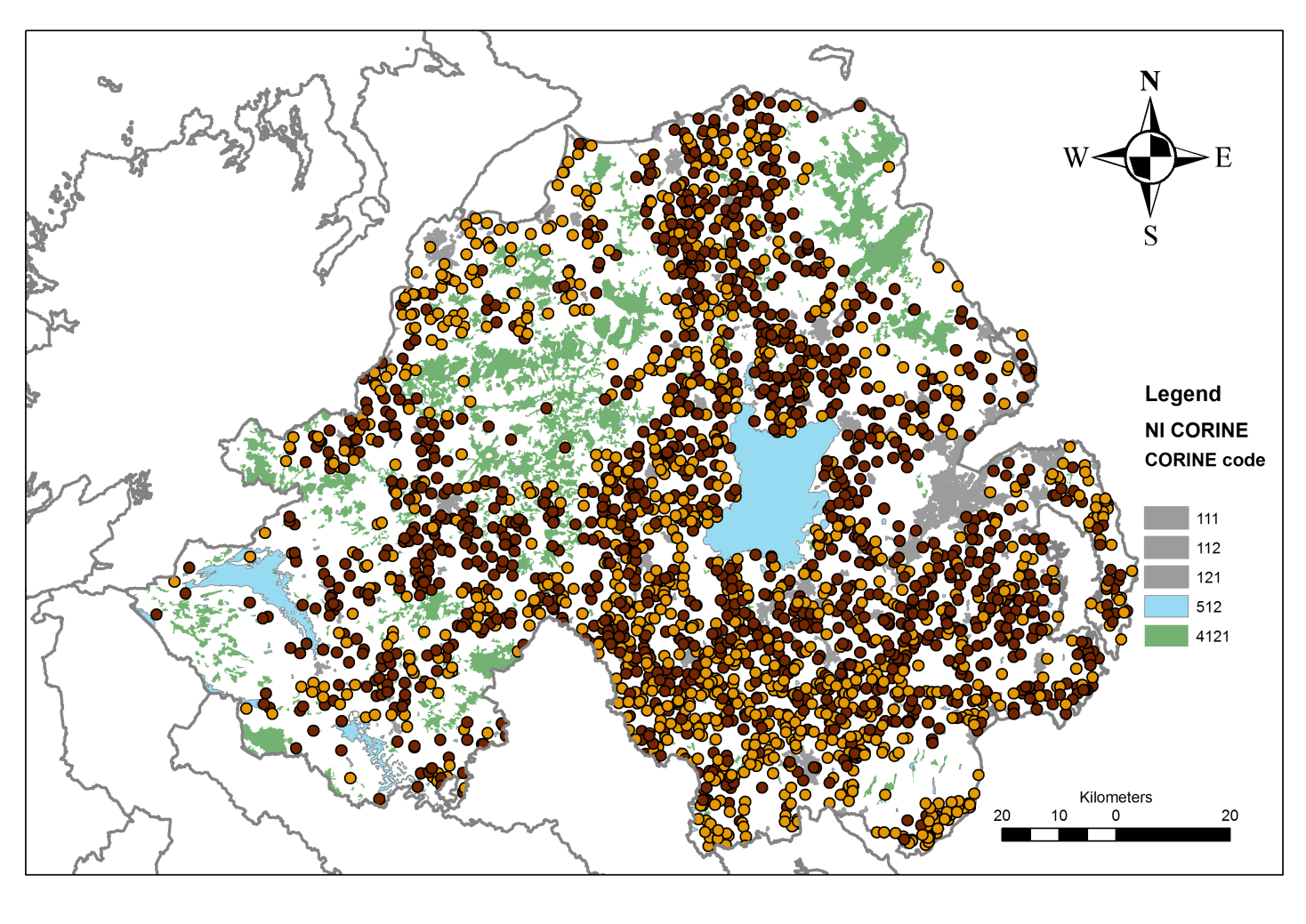


**Figure S7:** Point map of the severely infected (status = 1) farms and the non-severely fluke infected farms (status = 0) in Northern Ireland in 2013. The CORINE land cover types presented in this map corresponds with: 111=Continuous urban fabric; 112 = Discontinuous urban fabric; 121 = Industrial and commercial units; 512 = Water bodies; 4121 = Unexploited bogs.


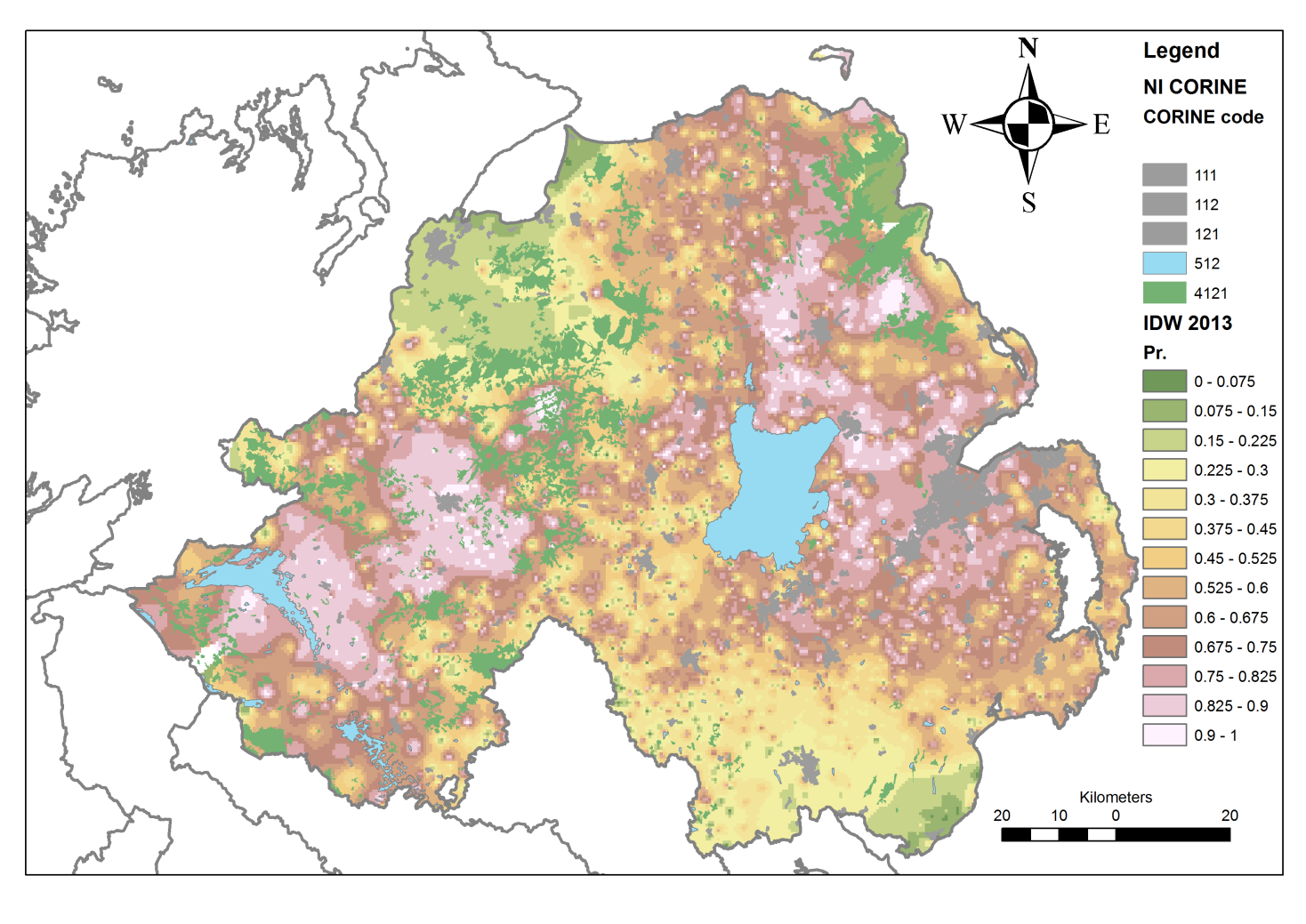


**Figure S8:** Raster map of the predicted within herd prevalence in Northern Ireland in 2013. The CORINE land cover types presented in this map corresponds with: 111=Continuous urban fabric; 112 = Discontinuous urban fabric; 121 = Industrial and commercial units; 512 = Water bodies; 4121 = Unexploited bogs.


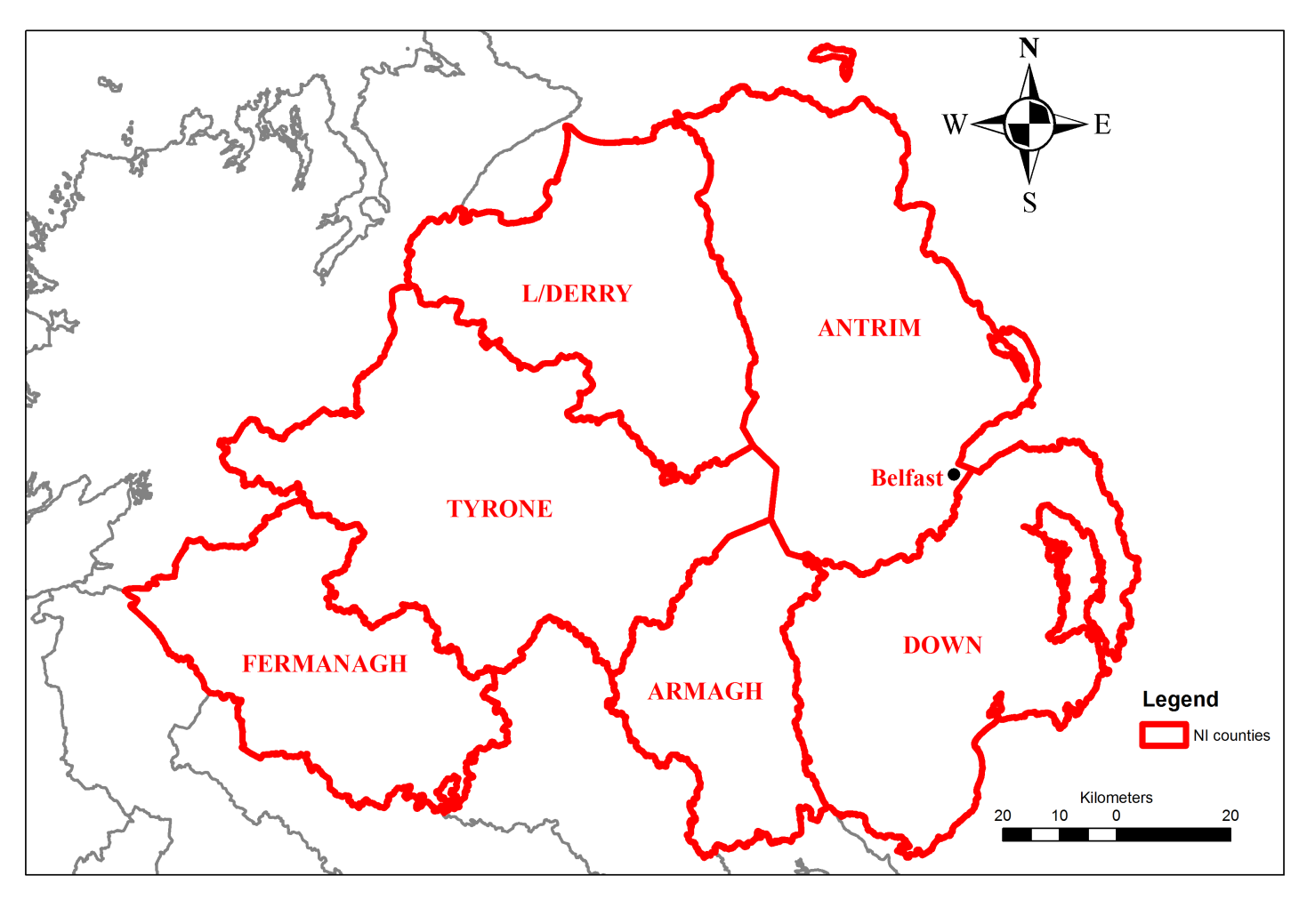


**Figure. S9:** Location of counties in Northern Ireland.


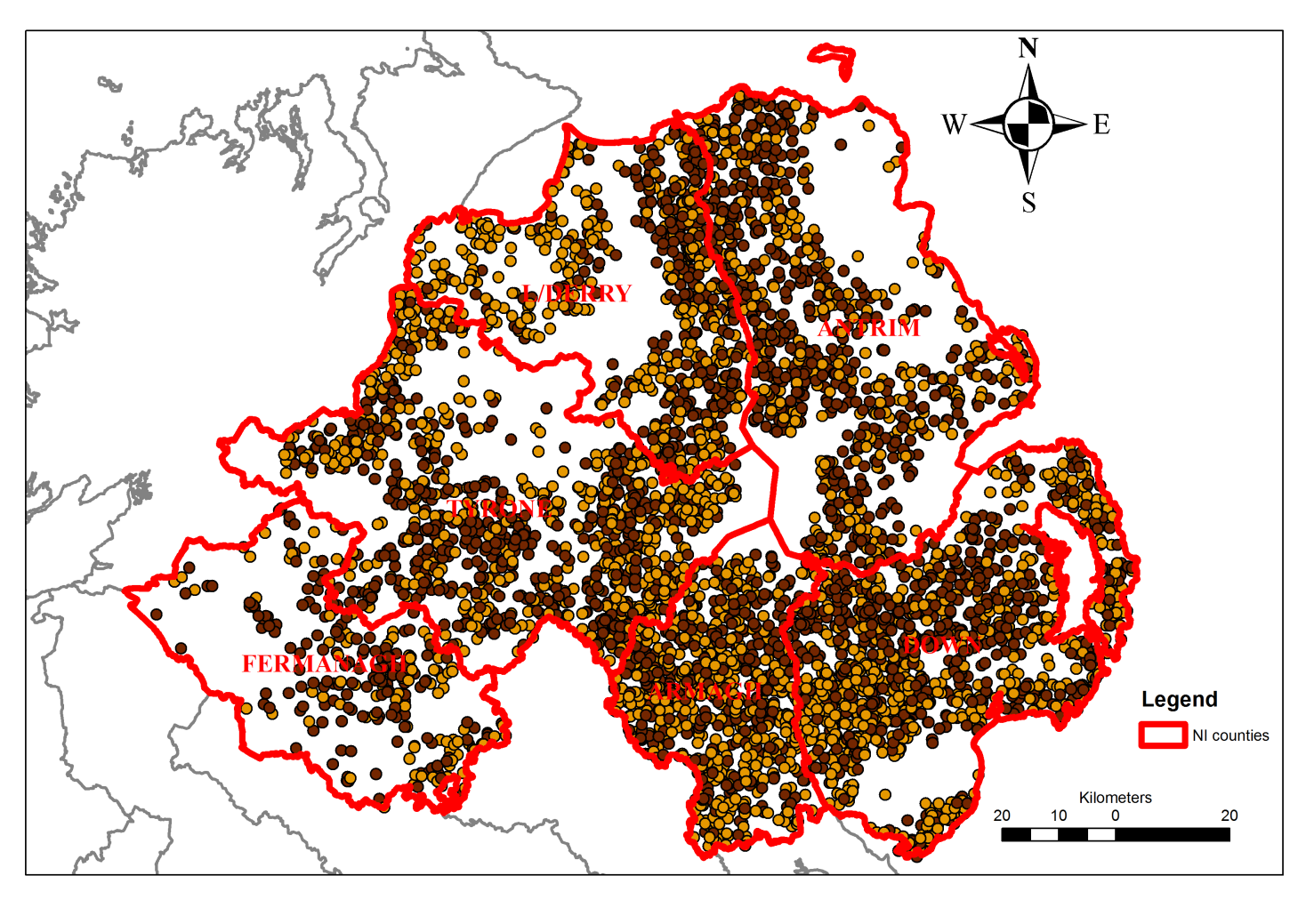


**Figure S10:** Location of all herds used in the binary logit model.

**Figure S11:** The relationship between the predicted within herd prevalence and the observed within herd prevalence from a generalised linear model (GLM) modelling the proportion of animals with evidence of fluke infection at slaughter in 2011 in Northern Ireland. The dashed line represents the line of perfect calibration.


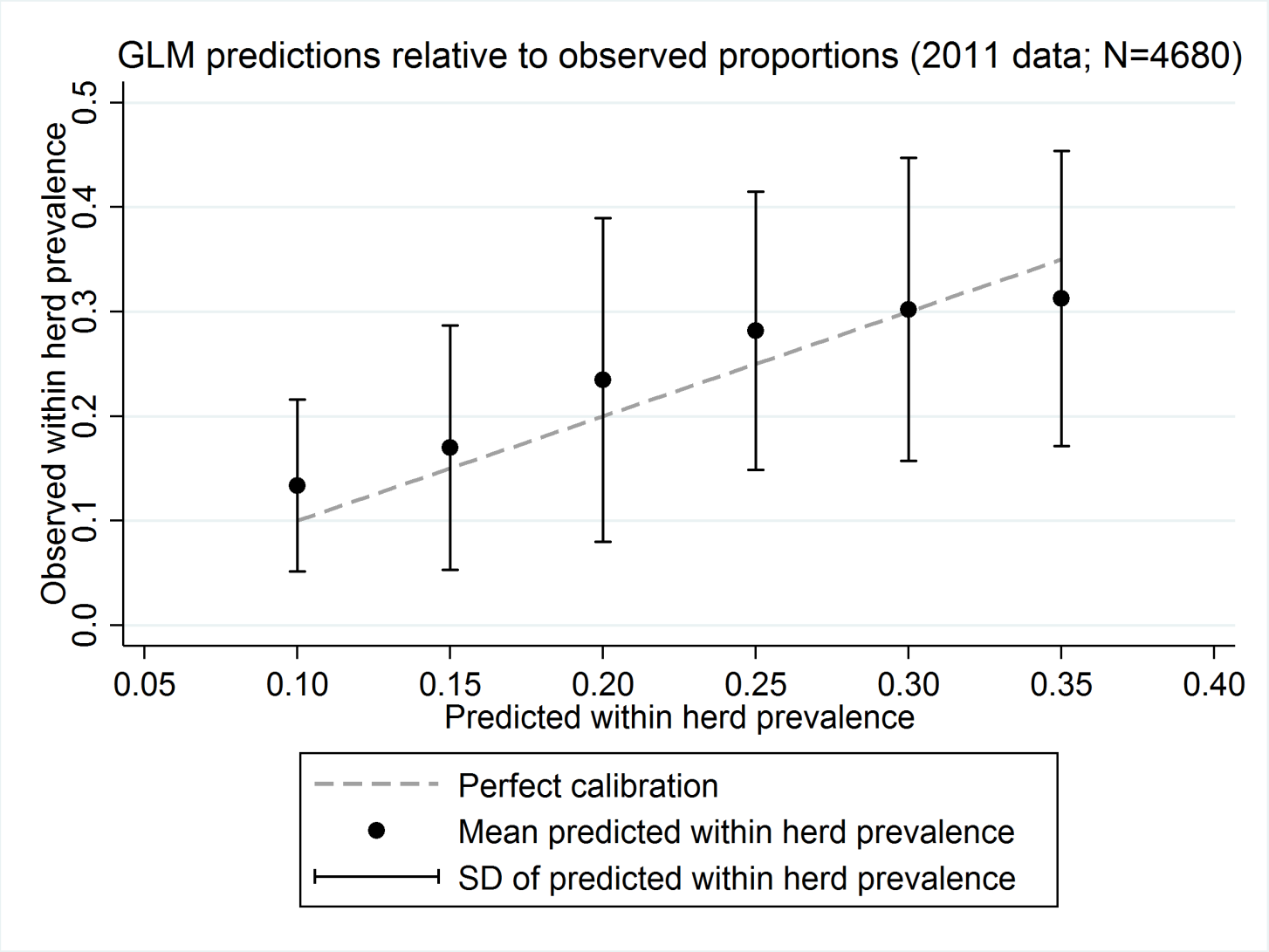


**Figure S12:** The relationship between the predicted within herd prevalence and the observed within herd prevalence from a generalised linear model (GLM) modelling the proportion of animals with evidence of fluke infection at slaughter in 2012 in Northern Ireland. The dashed line represents the line of perfect calibration.


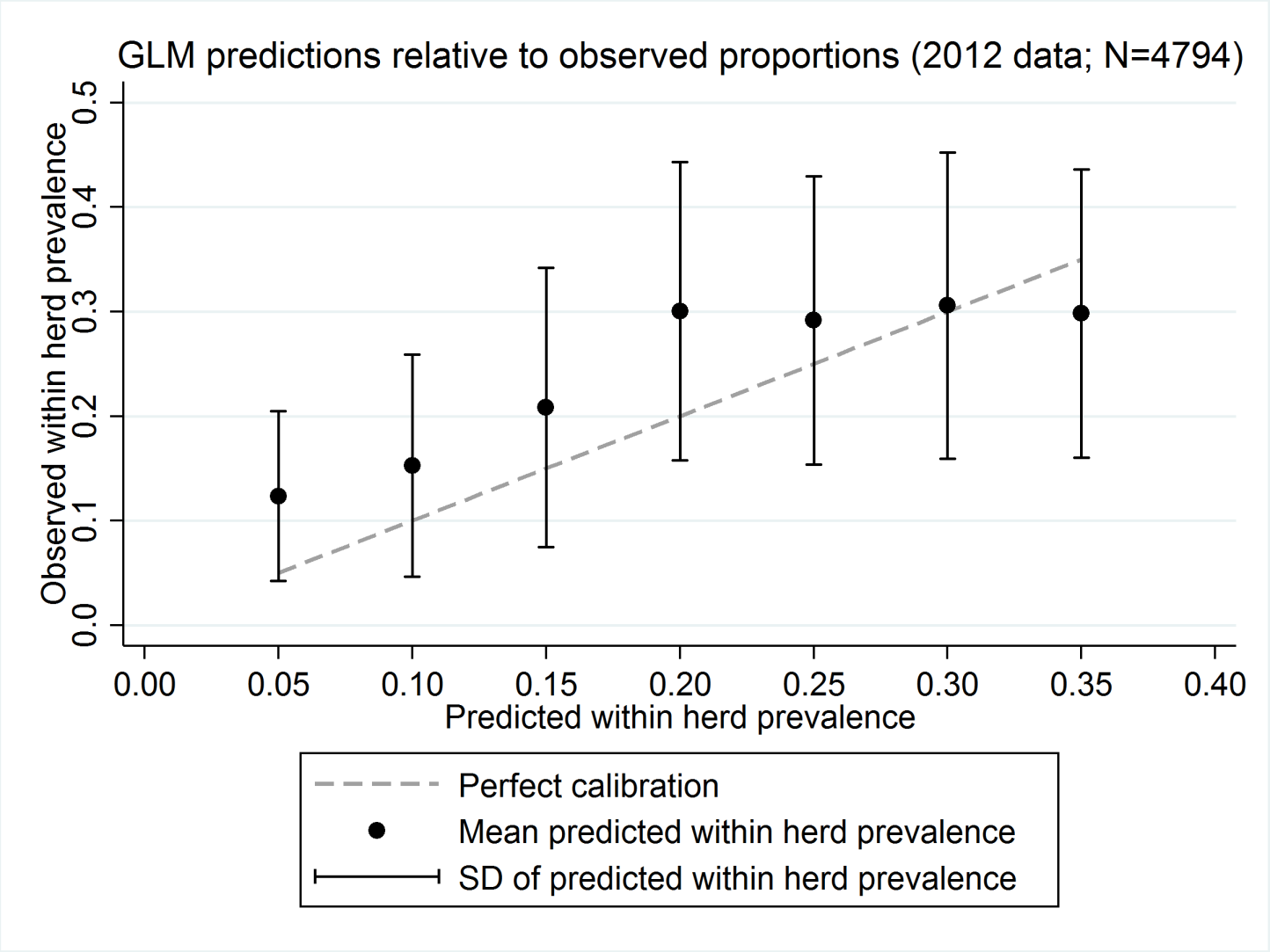


**Figure S13:** The relationship between the predicted within herd prevalence and the observed within herd prevalence from a generalised linear model (GLM) modelling the proportion of animals with evidence of fluke infection at slaughter in 2013 in Northern Ireland. The dashed line represents the line of perfect calibration.


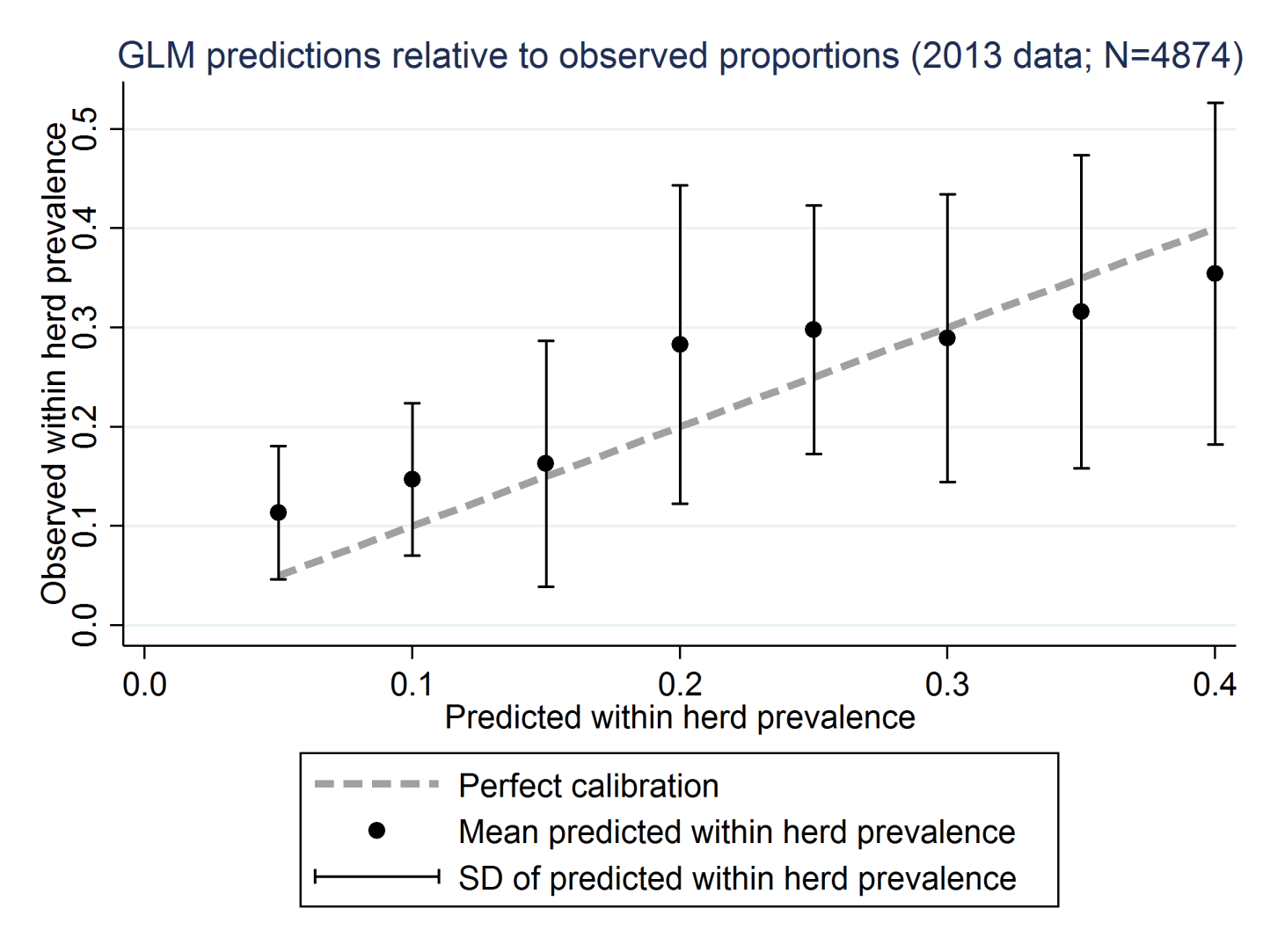

Supplement: Additional file 1: — Summary of predictor variables (Table S1-S5), example of spatial distribution of residuals and semi-variogram (Figures S1-S2), distribution and prediction maps (Figures S3-S10), and calibration graphs for GLM models (Figure S11-S13). (DOCX 6797 kb) [file 13071_2016_1489_MOESM1_ESM.docx]
